# Spontaneous recanalization of occluded transplant renal artery: a rare case report

**DOI:** 10.1186/s12882-020-02105-z

**Published:** 2020-10-19

**Authors:** Xiangjun Dong, Yingliang Wang, Huimin Liang, Chuansheng Zheng, Hui Zhao, Hong yao Hu, Xi Long, Yangbo Su

**Affiliations:** 1 Department of Radiology, Union Hospital, Tongji Medical College, Huazhong University of Science and Technology, Wuhan, 430022 China; 2 Hubei Key Laboratory of Molecular Imaging, Wuhan, 430022 China; 3 Department of Interventional Radiology, Renmin Hospital of Wuhan University, Wuhan, 430060 China

**Keywords:** Transplant renal artery stenosis, Kidney transplantation, Endovascular treatment, Stent, Case report

## Abstract

**Background:**

Transplant renal artery stenosis (TRAS) is a serious vascular complication that occurs after renal transplantation and can result in hypertension, renal functional impairment, and graft loss. Endovascular treatment has become the first-line treatment for TRAS because of its low invasiveness and high success rate.

**Case presentation:**

A 23-year-old female with end-stage renal disease of unknown cause received a living-donor kidney transplantation 10 months ago. Seven months after the transplantation, her blood pressure gradually deteriorated. Magnetic resonance angiography revealed bending and stenosis of the transplant renal artery, and the patient received endovascular treatment. A digital subtraction angiography revealed significant stenosis of 95% in the proximal transplant renal artery. The guidewire could not pass through the stenotic segment of the transplant renal artery even with repeated attempts by the surgeons; as a result, the transplant renal artery became occluded, and vasodilators were ineffective. After the operation, renal function gradually worsened, so she began to receive regular dialysis. Twenty-five days later, the patient’s urine volume was significantly higher than that before, and ultrasound showed that the proximal transplant renal artery was not completely occluded. A re-intervention was performed, and the stent was placed successfully in the stenotic segment. After the operation, renal function gradually recovered, and dialysis was no longer needed.

**Conclusion:**

Patients with iatrogenic transplant renal artery occlusion may have the possibility of spontaneous recanalization, which can help prevent the need for re-transplantation.

## Background

Transplant renal artery stenosis (TRAS) is a serious vascular complication that occurs after renal transplantation, and it can result in hypertension, renal functional impairment, and graft loss [1]. The incidence of TRAS is approximately 1 to 23% [2]. Endovascular treatment has become the first-line therapy for TRAS because of its low invasiveness and high success rate [3, 4]. Herein, we report a case of TRAS in which the stenotic segment was iatrogenically occluded on the first endovascular treatment attempt, followed by subsequent spontaneous recanalization and successful stent placement.

## Case presentation

**Fig. 1:**
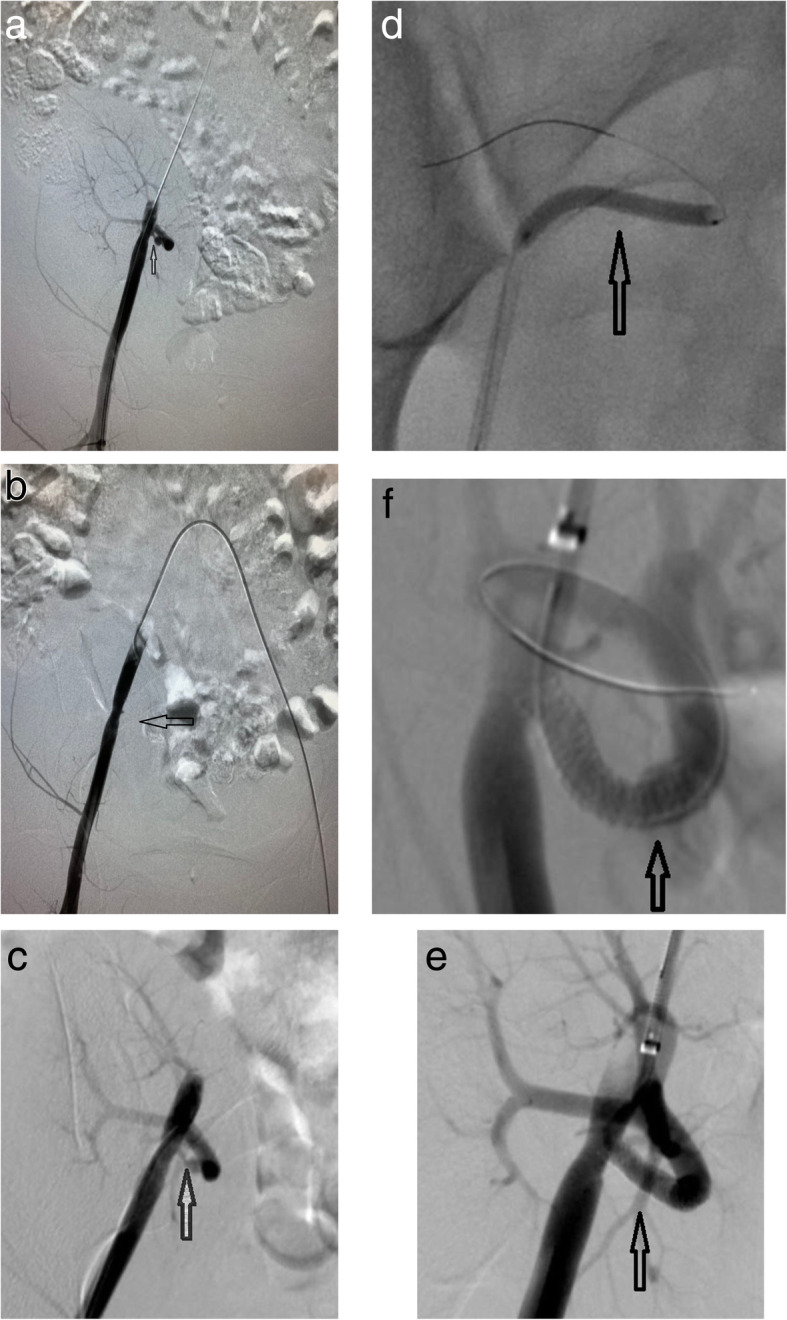
**a** and **b** show DSA images of the patient during the first endovascular treatment, demonstrating significant stenosis of 95% in the proximal transplant renal artery and transplant renal artery occlusion for repeated manipulations. **c** shows an angiographic image 25 days after the first operation, demonstrating spontaneous recanalization of the transplant renal artery. **d** shows a DSA image of the patient during the second endovascular treatment, demonstrating that a 3 mm × 30 mm balloon (Boston ultra-soft SV) was used to dilate the stenotic segment. **e** and **f** show DSA images of the patient, demonstrating that a 4 mm × 23 mm stent (Firehawk Rapamycin Target Eluting Coronary Stent) was implanted successfully in the stenotic segment and that the transplant renal artery was well reconstructed

A 23-year-old female with end-stage renal disease of unknown cause received a living-donor kidney transplantation 10 months ago, in which an end-to-side anastomosis of the donor renal artery to the patient’s right external iliac artery was performed. After the transplantation, her renal function was stable with an estimated glomerular filtration rate of approximately 75 ml/min/1.73 m^2^. Seven months after the transplantation, her blood pressure gradually deteriorated, and there was no other remarkable medical history. Magnetic resonance angiography was performed in another hospital, and it revealed a bending and stenosis of the transplant renal artery. On the day of admission to our hospital, her serum creatinine was 140.1 μmol/l. After obtaining the patient’s consent, endovascular treatment was attempted under the guidance of digital subtraction angiography. A 5-French Berenstein catheter (Cook) was inserted into the right external iliac artery via the right femoral artery, and angiography revealed significant stenosis (the degree of stenosis was approximately 95%, and the range of stenosis was approximately 20 mm) in the proximal transplant renal artery (Fig. 1a). An 0.014-in. guidewire (Cook) was used to pass through the stenotic segment through the right femoral artery pathway but failed; then we attempted to pass the guidewire through the stenotic segment through the left femoral artery pathway but failed again. A 5-French Berenstein catheter (Cook) was then placed at the opening of the right external iliac artery for angiography, which showed that the transplant renal artery was not opacified at all. The surgeons believed that the frequent endovascular manipulations may have stimulated the transplant renal artery resulting in an arterial spasm. Vasodilators (papaverine 30 mg plus normal saline 30 ml and nitroglycerin 0.2 mg plus normal saline 10 ml) were administered by slow infusion into the opening of the transplant renal artery. An angiography was performed 10 min later and showed that the transplant renal artery was still invisible which indicates the possibility of complete occlusion (Fig. 1b). Then, the operation was stopped, and the patient was returned to the ward and received intensive care. After the operation, the patients’ serum creatinine increased (up to 639.8 μmol/l) and urine volume decreased (less than 100 ml) gradually. Urologists were consulted. They advised that the patient should receive another kidney transplantation operation or regular dialysis because of the serious adhesion between the transplanted kidney and the surrounding tissue after the first transplant operation. As a result, the patient began to receive regular dialysis (3 times per week) and no other medical therapy was offered.

Twenty-five days after the operation, the patient’s urine volume began to increase gradually, and then an ultrasound examination was performed which revealed that the proximal segment of the transplant renal artery was stenotic and the distal segment was patent. The patient was hospitalized and underwent endovascular treatment again. The preoperative serum creatinine was 335 μmol/l. After obtaining consent, the endovascular procedure was performed under the guidance of digital subtraction angiography (DSA). First, a 5-French angiographic catheter (Cook) was used to perform an angiography at the opening of the right external iliac artery through the right femoral artery, which showed that the proximal stenosis of the transplanted renal artery was approximately 95%, and the distal segment was unobstructed (Fig. 1c). Second, an 0.014-in. guidewire (Cook) was used to pass through the stenotic segment through the right femoral artery access successfully. Then, a 3 mm × 30 mm balloon (Boston ultra-soft SV) was used to dilate the stenotic segment (Fig. 1d), but the extent of stenosis was still greater than 50% after the dilatation. Subsequently, the surgeon attempted to implant a 4 mm × 23 mm stent (Firehawk rapamycin targeted eluting stent system) into the stenotic segment, but the stent system could not be accurately placed in the stenotic segment. After that, the stent delivery system was withdrawn, and left femoral artery access was established. The stent delivery system arrived in the stenotic segment, and the stent was eventually successfully released (Fig. 1e and Fig. 1f). After the operation, the patient’s renal function gradually improved, serum creatinine gradually decreased (102 μmol/l at the time of discharge), urine volume returned to normal, and she was able to discontinue dialysis.

## Discussion and conclusion

TRAS is an important cause of poor long-term prognosis and low survival rate of kidney transplantation [5]. Its clinical manifestations are atypical and mainly include refractory hypertension and graft dysfunction, or it is asymptomatic [1]. The incidence of TRAS is 1 to 23% [2].

Timely and effective imaging examination is critical for the early diagnosis of TRAS. Ultrasonic Doppler has been recommended as the first-line screening method because of its high sensitivity, specificity, and cost-effectiveness [6]. At present, the commonly used diagnostic standard is systolic peak velocity (SPV) > 200 mm/s [2]. However, the obvious disadvantage of ultrasound examination is that diagnosis is significantly influenced by the operator’s technical experience. Computed tomographic angiography (CTA) has a high spatial resolution, and its three-dimensional post-processing function can directly display the stenosis site and enables evaluation of the degree of stenosis and the condition of adjacent tissues from multiple angles. Moreover, with the use of non-ionic contrast agents, CTA damage to renal function is also significantly reduced [7]. Magnetic resonance angiography (MRA) is often used in patients with severe renal dysfunction. It has high sensitivity and specificity, and the contrast agent is not toxic to the kidney [8]. However, it has been shown that MRA tends to overestimate the extent of stenosis [7]. Digital subtraction angiography (DSA) is still considered the gold standard for TRAS diagnosis and can be used for endovascular treatment [2]. However, it is only used during endovascular operation to treat TRAS because of the large dosage of contrast agent required. According to our experience and existing literature reports, ultrasound should be considered the first-line screening method for diagnosing TRAS, and regular follow-up should be carried out for patients with kidney transplant; for those suspected to have TRAS by ultrasound, CTA or MRA should be performed for further diagnosis; then DSA should be performed to confirm the diagnosis and perform the treatment.

Endovascular treatment of TRAS is considered the first-line therapeutic method in most centres [3, 9]. A systematic review published by Ngo et al. [2] showed that the average technical success rate of endovascular treatment was 93.7%, and the average restenosis rate was 14.7%. In our experience, the balloon-expandable stent has good radial force and can be positioned accurately, which makes it more suitable for short and straight stenotic lesions; the self-expanding stent has good flexibility and is more suitable for curved stenotic lesions. In recent years, drug-eluting stents have been used to treat TRAS. The drug-eluting stent can inhibit the proliferation of intima and reduce the re-stenosis rate [10]. In this case, we used the Firehawk Rapamycin Target Eluting Stent because of its advantages in radial force, stent flexibility and eluting capacity.

In this case, the first attempt to treat TRAS failed. Twenty-five days later, the occluded vessel exhibited spontaneous recanalization. It can be assumed that the repeated manipulations during the first treatment caused severe spasm of the wall of a narrowed artery, oedema, and even intramural haematoma, eventually leading to complete closure of the vessel. After some time, iatrogenic occlusion of the artery was self-repairing, the transplanted kidney suffered acute kidney injury, and the next endovascular procedure was effective. This case alerts us that patients with iatrogenic transplant renal artery occlusion may have the possibility of spontaneous recanalization, which can help prevent the need for re-transplantation. Similarly, practitioners should not treat the patient’s existing condition (in various clinical situations) as permanent and irreversible. Careful supervision and reaction to an ever-changing clinical situation may be a beneficial solution for the patient.

## Data Availability

All data generated or analysed during this study are included in the published article.

## Abbreviations

TRAS: Transplant renal artery stenosis
CTA: Computed tomographic angiography
DSA: Digital subtraction angiography
MRA: Magnetic resonance angiography

## References

[1] Kadoya Y, Zen K, Matoba S (2018). Endovascular treatment of transplant renal artery stenosis based on hemodynamic assessment using a pressure wire: a case report. Bmc Cardiovasc Disor.

[2] Ngo AT, Markar SR, De Lijster MS, Duncan N, Taube D, Hamady MS (2015). A systematic review of outcomes following percutaneous Transluminal angioplasty and stenting in the treatment of transplant renal artery stenosis. Cardiovasc Inter Rad.

[3] Braga AFF, Catto RC, Dalio MB, Tenório EJR, Ribeiro MS, Piccinato CE (2015). Endovascular Approach to Transplant Renal Artery Stenosis. Ann Transpl.

[4] Valle LG, Cavalcante RN, Motta-Leal-Filho JM, Affonso BB, Galastri FL, Doher MP (2017). Evaluation of the efficacy and safety of endovascular management for transplant renal artery stenosis. Clinics.

[5] Biederman DM, Fischman AM, Titano JJ, Kim E, Patel RS, Nowakowski FS (2015). Tailoring the endovascular Management of Transplant Renal Artery Stenosis. Am J Transplant.

[6] Fananapazir G, McGahan JP, Corwin MT, Stewart SL, Vu CT, Wright L (2017). Screening for Transplant Renal Artery Stenosis: Ultrasound-Based Stenosis Probability Stratification. AJR Am J Roentgenol.

[7] Zhang X, Wang H, Liu S, Yan J, Liu X, Xu D (2017). Three-dimensional computed tomography reconstruction in transplant renal artery stenosis. Exp Clin Transplant.

[8] Bley TA, François CJ, Schiebler ML, Wieben O, Takei N, Brittain JH (2016). Non-contrast-enhanced MRA of renal artery stenosis: validation against DSA in a porcine model. Eur Radiol.

[9] Li X, Zhang J, Meng Y, Yang L, Wang F, Li B, et al. Transplant renal artery stenosis caused by the stretch of an artey branch: a case report and literature review. BMC Nephrol 2018;19(1):56. 10.1186/s12882-018-0856-y PMC5845193 29523086

[10] Estrada CC, Musani M, Darras F, Suh H, Abate MT, Mani A (2017). 5 years experience with drug eluting and bare metal stents as primary intervention in transplant renal artery stenosis. Transplantation Direct.

